# Impaired neutrophil extracellular trap-forming capacity contributes to susceptibility to chronic vaginitis in a mouse model of vulvovaginal candidiasis

**DOI:** 10.1128/iai.00350-23

**Published:** 2024-01-30

**Authors:** Junko Yano, Paul L. Fidel

**Affiliations:** 1 Department of Oral and Craniofacial Biology, Louisiana State University Health, New Orleans, Louisiana, USA; University of California, Davis, Davis, California, USA

**Keywords:** *Candida albicans*, vulvovaginal candidiasis, mouse model, neutrophil extracellular traps, inflammation

## Abstract

Vulvovaginal candidiasis (VVC), caused by *Candida albicans,* is characterized by aberrant inflammation by polymorphonuclear neutrophils (PMNs) in the vaginal lumen. Data from the established murine model shows that despite potent antifungal properties, PMNs fail to clear *C. albicans* due to local heparan sulfate that inhibits the interaction between PMNs and *C. albicans*, resulting in chronic vaginal immunopathology. To understand the role of neutrophil extracellular traps (NETs) in defense against *C. albicans* at the vaginal mucosa, we investigated the NET-forming capacity of PMNs in chronic VVC-susceptible (CVVC-S/C3H) and -resistant (CVVC-R/CD-1) mouse strains. Immunofluorescence revealed the formation of NETs (release of DNA with PMN-derived antimicrobial proteins) in PMN*–C. albicans* cocultures using vaginal conditioned medium (VCM) generated from CVVC-R/CD-1 mice, similar to NET-inducing positive controls. Under these NETotic conditions, PMNs released high levels of double-stranded DNA bound with NET-associated proteins, concomitant with substantial *C. albicans* killing activity. In contrast, PMN*–C. albicans* cocultures in VCM from CVVC-S/C3H mice lacked NET formation together with reduced antifungal activity. Similar results were observed *in vivo*: active NET*–C. albicans* interaction followed by fungal clearance in inoculated CVVC-R/CD-1 mice, and sustained high vaginal fungal burden and no evidence of NETs in inoculated CVVC-S/C3H mice. Furthermore, the level of Ki67 expression, a putative NETotic PMN marker, was significantly reduced in vaginal lavage fluid from CVVC-S/C3H mice compared to CVVC-R/CD-1 mice. Finally, scanning electron microscopy revealed that PMNs in CVVC-R, but not CVVC-S, conditions exhibited NETs in direct contact with *C. albicans* hyphae *in vitro* and *in vivo*. These results suggest that VVC-associated immunopathology includes impaired NET-mediated antifungal activity.

## INTRODUCTION

Vulvovaginal candidiasis (VVC) is a common fungal infection caused by an opportunistic pathogen *Candida albicans* (1). VVC affects approximately 75% of women at least once during their reproductive years, with an additional 5%–8% experiencing recurrent VVC (RVVC), defined as three or more episodes per year (2, 3). Symptoms of VVC and RVVC include itching, burning, redness of the vulva and vaginal mucosa, and white vaginal discharge (4, 5). The current antifungal therapies provide only short-term relief by reducing fungal burden and do not offer a complete cure. Relapses are common despite the absence of known predisposing factors (hormonal changes, disturbance in microbiome composition, and uncontrolled diabetes mellitus) and necessitate maintenance of antifungal regimens (3). VVC and RVVC negatively impact the quality of life for otherwise healthy women and impose a substantial healthcare burden worldwide.

Early research had focused on T cell-mediated immunity as a potential mechanism underlying susceptibility to VVC/RVVC, similar to other forms of *Candida* infections (6). However, investigations into local or systemic adaptive immunity, including cytokine or antibody responses, did not identify a protective role in vaginitis (7–12). Instead, an aberrant inflammatory immunopathogenic response by polymorphonuclear neutrophils (PMNs) has been implicated as a hallmark of susceptibility to symptomatic vaginitis in both women and the estrogen-dependent mouse model (13–17). Accordingly, subsequent work in the mouse model demonstrated that PMNs were unable to effectively clear *Candida* in most inbred mouse strains (e.g., C3H and C57BL/6) due to the presence of vaginal heparan sulfate (HS) that blocks PMN*–C. albicans* interactions. This state of pathogenicity was defined as a chronic VVC-susceptible (CVVC-S) or symptomatic condition. In contrast, CD-1 mice that are inherently hypo-responsive to exogenous estrogen and with a low presence of putative HS are capable of clearing vaginitis through effective PMN antifungal activity and, thus, considered chronic VVC-resistant (CVVC-R) (15–20). To date, however, the details surrounding the function of PMNs underlying fungal clearance during experimental vaginitis have not been characterized.

PMNs primarily kill pathogens through phagocytosis (engulfment of invading microorganisms), degranulation (release of antimicrobial agents from granules), and formation of neutrophil extracellular traps (NETs, entrapment of extracellular microbes) (21). NETs are web-like structures that consist of chromosomal DNA fibers from PMNs bound with histones, granular and cytoplasmic components that possess antimicrobial properties (22, 23). The fibrous structures of NETs allow direct entrapment or can act as physical barriers against large extracellular microbes, such as *C. albicans* hyphae. Furthermore, NETs can serve as vehicles that enable pathogen-targeted delivery of antimicrobial molecules, such as neutrophil elastase (NE) and myeloperoxidase (MPO), at a high local concentration (24, 25). The process of NET formation is known as NETosis during which disassembly of the nucleus and granules occurs followed by plasma membrane rupture, release of cell contents, and ultimately cell death (22, 26). Recently, several proteins involved in the cell cycle pathway have been proposed as markers of NETotic PMNs including Ki67, a nuclear protein involved in cell proliferation (27). While PMNs are terminally differentiated leukocytes, evidence suggests that certain proteins associated with cell division are repurposed for the induction of chromatin decondensation and nuclear envelope rupture that similarly occur during mitosis and NETosis. NETs have been implicated in a variety of conditions involving fungal pathogens, such as *Candida* spp., *Aspergillus fumigatus*, and *Histoplasma capsulatum* (28–30). Previous studies have shown that both yeast and hyphal forms of *Candida* spp. stimulated NETosis *in vitro* and *in vivo* (23). However, little is known regarding the involvement of NET formation during vaginal infection. A study reported that the number of NETotic cells in vaginal discharge of women with VVC was significantly increased compared to those without infection, albeit at a much lower level than samples from women with *Trichomonas vaginalis* infection (31). Given that competent PMNs fail to reduce vaginal fungal burden despite their robust migration to the vaginal cavity during infection, we hypothesize that the lack of *Candida* clearance is associated with impaired NET formation in the vaginal mucosa, resulting in chronic infection and immunopathology. Therefore, the objective of this study was to conduct comparative evaluations of NET-forming capacities by employing *in vitro* and *in vivo* models of CVVC-S and CVVC-R conditions.

## MATERIALS AND METHODS

### Mice

Female C3H and CD-1 mice (5–7 weeks old) were purchased from Charles River Laboratories. The mice were housed and handled in AAALAC-approved facilities located in the LSU Health School of Dentistry. All animal protocols were reviewed and approved by the Institutional Animal Care and Use Committee of LSU Heath, New Orleans, LA. The mice were provided with standard chow and water *ad libitum* and monitored daily for signs of distress.

### Microorganisms

*C. albicans* ATCC 96113, a vaginal isolate, was used throughout the study unless otherwise specified. For scanning electron microscopy (SEM) analysis, *in vitro* cultures were performed using *C. albicans* SC5314 and 96113 in parallel. Both strains of *C. albicans* were maintained in a 20% glycerol stock medium at −80°C. A 10-µL aliquot from the glycerol stock was streaked onto yeast extract–peptone–dextrose (YPD) agar and cultured at 35°C for 48 h. A single colony was transferred into 10 mL YPD broth and incubated at 30°C for 18 h with shaking at 200 rpm until the culture reached the stationary phase. Following incubation, *C. albicans* cells were washed three times in sterile phosphate-buffered saline (PBS) and enumerated using a hemocytometer.

### Murine model of chronic vulvovaginal candidiasis

Intravaginal inoculation of *C. albicans* in C3H (CVVC-susceptible) and CD-1 (CVVC-resistant) mice was conducted as previously described (16, 32). Briefly, mice were subcutaneously injected with 0.1 mg β-estradiol 17-valerate (Sigma) dissolved in 100 µL sesame oil 72 h prior to inoculation. Injections were repeated weekly as needed. Estrogen-treated mice were intravaginally inoculated with 20 µL of PBS containing *C. albicans* 96115 (5 × 10^4^) blastoconidia into the vaginal lumen. At specific time points post-inoculation, vaginal lavage was performed under anesthesia by isoflurane inhalation. Briefly, 100 µL of PBS was introduced into the vaginal lumen and aspirated several times with gentle agitation using a pipette tip. The resulting lavage fluids were transferred individually into 0.6-mL microcentrifuge tubes and processed for confocal microscopy or SEM analyses.

### Vaginal conditioned medium

Vaginal lavage fluids from estrogen-treated, uninoculated C3H or CD-1 mice were collected as previously described except using a 100 µL RPMI 1640 medium instead of PBS (16). Lavage fluids were pooled from 5 to 10 mice per strain and centrifuged at 200 *g* for 5 min. Supernatants were filtered with a 0.45-µm-pore-size syringe filter and stored at −80°C until use.

### PMN isolation

Elicited murine peritoneal PMNs were obtained from peritoneal exudates harvested 12 h post-intraperitoneal injection of 2 mL 10% casein sodium in PBS. PMNs were enriched by hypotonic lysis of erythrocytes and washed three times in sterile PBS. Viable PMNs were identified by trypan blue dye exclusion and enumerated using a hemocytometer. The final enrichment of PMNs, ranging from 85% to 95% Gr-1/Ly6G^+^ cells, has been confirmed previously by flow cytometry (14). In each *in vitro* experiment, PMNs were isolated from naïve mice, and this source of PMNs was used for individual sets of cocultures.

### PMN*–C. albicans* cocultures and killing assay

Fungal killing capacities of PMNs *in vitro* were assessed using a *C. albicans*–PMN coculture assay as previously described (16). PMNs resuspended at 5 × 10^6^/mL in a RPMI 1640 medium were transferred to a 96-well plate in a volume of 100 µL per well and preincubated for 30 min at 37°C with 5% CO_2_ to obtain a monolayer. *C. albicans* blastoconidia (5 × 10^5^/mL) were opsonized with 5% mouse serum for 30 min at room temperature. Following the initial incubations, PMN monolayers (5 × 10^5^/well) were inoculated with *C. albicans* 96113 (5 × 10^4^/well) in a volume of 100 µL. After incubation for 30 min at 37°C with 5% CO_2_, unbound *C. albicans* cells were removed by gently washing the well with PBS. Following washing, the cocultures were incubated in 100 µL of RPMI 1640 or vaginal conditioned medium (VCM) alone or VCM supplemented with purified HS (Sigma, 10–400 µg/mL), heparanase (HPSE, Sigma, 5 U), or phorbol 12-myristate 13-acetate (PMA) (100 nM) for 3 h at 37°C with 5% CO_2_. *C. albicans* were cultured alone in each respective medium as controls.

Following the final incubation of PMN*–C. albicans* coculture, the wells were washed twice with 100 µL of wash buffer (0.05% Triton X in water) to lyse PMNs and harvest *C. albicans* cells. The numbers of viable *C. albicans* were quantified by plate counts of CFUs after incubation for 24 h at 35°C. The percent killing was assessed in comparison with *C. albicans* cultured alone in each respective vehicle and calculated as follows: % killing = (1 – CFU from coculture with PMNs/CFU from *C. albicans* cultured alone) × 100. Results were expressed as % killing ± standard errors of the mean (SEM).

### Quantification of extracellular DNA release

PMN*–C. albicans* cocultures were performed as previously described using VCM. Controls included PMN*–C. albicans* cocultures in RPMI 1640 alone or the vehicle supplemented with 100 nM PMA (Sigma). Following the 3-h incubation of the cocultures, 100 µL of PBS containing 0.5 U of micrococcal nuclease (MNase, New England Biolabs) was added to the wells and incubated for 10 min at 37°C to release extracellular DNA from the cocultures. Supernatants were collected and assayed for concentrations of double-stranded DNA (dsDNA) using the Quant-iT PicoGreen dsDNA assay kit (Life Technologies) according to the manufacturer’s instruction. Alternatively, the release of dsDNA was detected and quantified using a cell impermeable SYTOX staining technique. Following the 3-h incubation of cocultures, wells were gently washed with Hanks’ Balanced Salt Solution (HBSS) and incubated with 100 µL of HBSS containing 0.5 µM SYTOX Green nucleic acid stain (Thermo Fisher Scientific) for 15 min at room temperature in the dark. Fluorescence was measured at 504/523 nm using a Synergy microplate reader (Bio-Tek). Results were expressed as picogram per milliliter (pg/ml) (the PicoGreen assay), or relative fluorescence intensity (RFI) (the SYTOX assay), ± SEM.

### Protease activity assay

Supernatants of vaginal lavage fluids from C3H and CD-1 mice collected at 0, 24, 48, and 96 h post-inoculation were evaluated for proteolytic activity using a Pierce Fluorescent Protease activity assay kit (Thermo) designed for detecting the level of digested FITC-labeled casein as a measurable substrate. Resultant values were normalized to the protein content (per milligram) of each sample measured by the Pierce BCA protein assay reagent kit (Thermo). The proteolytic activity of trypsin (1 µg) was measured in parallel and used as a positive control. Results were expressed as RFI/mg protein ± SEM.

### Immunofluorescent staining

For visualization of NET markers *in vitro,* the standard PMN*–C. albicans* coculture assay was performed using 16-well Nunc Lab-TeK chamber glass slides (Thermo). Cocultures of PMN (5 × 10^5^/well) and *C. albicans* (5 × 10^4^/well) in a volume of 100 µL were prepared using RPMI 1640 alone or experimental VCM and incubated for 2–4 h at 37°C with 5% CO_2_. Controls included PMNs cultured in RPMI 1640 alone or with PMA in the absence of *C. albicans*. For the detection of NET markers *in vivo,* vaginal lavage fluids from inoculated mice at 2–4 days post-inoculation were diluted 10-fold in PBS and cytospun onto polysine-coated microscope slides using a Cytospin 4 cytocentrifuge (Thermo). Following coculture incubation or cytospun preparation, slides were fixed in 4% paraformaldehyde (Thermo) for 15 min and permeabilized with 0.5% Triton X-100. After washing, in PBS, slides were treated with a blocking buffer fetal bovine serum (5% FBS) and stained with primary antibodies [anti-MPO (2 µg/mL, Abcam), anti-NE (10 µg/mL, R&D Systems), anti-histone H3 (5 µg/mL, BioLegend), anti-Ly6G (1 µg/mL, R&D Systems), and anti-Ki67 (5 µg/mL, BioLegend)] overnight at 4°C. After washing, slides were further stained with fluorophore-conjugated secondary antibodies for 30 min, if necessary, followed by a DNA dye Hoechst 33258 (1 µg/mL, Thermo) for 15 min. Slides were examined using an Olympus FV100 confocal microscope with FluoView software.

### Gene expression analysis of *Ki67* by real-time PCR

Cell pellets of vaginal lavage fluids from inoculated mice were subjected to total RNA extraction using the QIAzol lysis reagent followed by purification using the RNeasy Minikit (Qiagen). Synthesis of cDNA from 10 ng of RNA was completed using the RevertAid H Minus First Strand cDNA synthesis kit (Thermo) according to the manufacturer’s instruction. Real-time PCR was performed using specific primers for mouse *Ki67* or *Act1* in conjunction with the PowerTrack SYBR Green master mix (Thermo) according to the manufacturer’s instructions. The PCR products were detected in 45 consecutive cycles (95°C for 15 sec and 60°C for 1 min) in an CFX Duet detection system and Maestro software (Bio-Rad). Signals of *Ki67* were normalized to those of a reference gene (*Act1*) and analyzed to quantify relative expression levels using the ΔΔC*_T_* method. The results are expressed as the fold increase over expression in lavage cells from uninoculated mice.

### Scanning electron microscopy

For visualization of NETs *in vitro*, PMN*–C. albicans* cocultures were performed on Nunc Thermanox coverslips (Thermo) placed in a 24-well plate. Cocultures of PMN (2 × 10^6^/well) and *C. albicans* 96113 or SC5314 (2 × 10^5^/well) in a volume of 500 µL were prepared using RPMI 1640 alone or experimental VCM and incubated for 4 h at 37°C with 5% CO_2_. Controls included PMNs cultured in RPMI 1640 alone or with PMA in the absence of *C. albicans*. For the evaluation of NETs *in vivo*, vaginae from inoculated mice were resected and opened into a sheet by making a lateral incision. Samples were fixed with primary fixation buffer [2.5% glutaraldehyde and 4% paraformaldehyde, Electron Microscopy Sciences (EMS)] for 1 h, washed, and post-fixed with 1% osmium tetroxide (EMS) for 1 h. Samples were rinsed with water and dehydrated with a graded ethanol series (25%–100% for 5 min each) and dried in hexamethyldisilazane (HMDS, EMS, 50% in ethanol then 100% HMDS for 15 min each). Samples were loaded onto aluminum studs with double-sided magnetic tapes and sputter-coated with carbon. Samples were imaged at 2,000×–10,000× magnifications on a Hitachi 4800 high-resolution electron microscope (Tulane University Coordinated Instrumentation Facility).

### Statistics

All experiments were conducted using 3–10 mice per group. All VCM samples prepared from vaginal lavage fluids were pooled using at least five mice per group. All data were analyzed for statistical significance using one-way analysis of variance (ANOVA) followed by *post hoc* unpaired Student’s *t*-test for comparisons made between the experimental and control groups. Significant differences were defined as a confidence level where the *P*-value was <0.05. All statistical results and graphs were generated using the GraphPad Prism software.

## RESULTS

### Differential NET-inducing capacities between CVVC-susceptible and -resistant conditions

**Fig 1:**
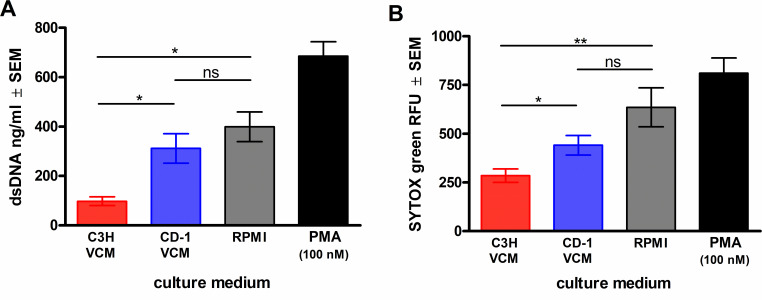
Quantification of DNA release by PMN*–C. albicans* cocultures in chronic VVC-susceptible and -resistant conditions *in vitro.* VCM was prepared by pooling vaginal lavage fluid from 5–10 estrogenized C3H (CVVC-susceptible) or CD-1 (CVVC-resistant) mice. Elicited peritoneal PMNs suspended in VCM or RPMI alone were preincubated to form monolayers and then cocultured with *C. albicans* 96113 cells in a specified culture medium or with PMA (100 nM) for 3 h. (**A**) Monolayers treated with MNase and the amount of dsDNA in the supernatants measured using a Quant-iT PicoGreen dsDNA assay. (**B**) Monolayers treated with SYTOX Green and measured for fluorescent intensity. Data were analyzed using one-way ANOVA among the RPMI control and the VCM groups followed by unpaired Student’s *t*-test to compare each VCM group with the control. Bar heights and error bars reflect the group mean ± SEM of % killing values computed from independent replicates of each of the four unique VCM samples. ^*^P < 0.05; ^**^P < 0.01. n.s., not significant; RFU, relative fluorescent unit.

To assess the relative capacities of PMNs to induce NET formation under chronic VVC-susceptible (CVVC-S) and -resistant (CVVC-R) conditions, we employed a standard PMN killing assay with modifications to simulate the vaginal environment *in vitro*. For this, RPMI-based VCM was generated using vaginal lavage fluid from CVVC-S (C3H) and CVVC-R (CD-1) mice, which contains native secretory factors present in the vaginal cavity. The levels of DNA release by PMNs, a primary indicator of NET formation, in response to a *C. albicans* challenge were evaluated in the coculture system utilizing VCM. Results showed extracellular dsDNA released during the 3 h coculture in VCM obtained from CVVC-R/CD-1 mice at similar levels to RPMI cultures or in a PMA-containing medium (a NET-inducing positive control) (Fig. 1A), whereas dsDNA release was significantly reduced in VCM from CVVC-S/C3H mice. As additional confirmation, PMN*–C. albicans* cocultures were evaluated for the release of extracellular dsDNA using a cell-impermeable DNA dye SYTOX Green. Fluorescent images of the non-permeabilized cocultures revealed SYTOX Green-positive PMNs in response to NET-inducing stimuli (*C. albicans* hyphae or PMA) starting at 1.5 h incubation and more intensely at 3 h. In contrast, minimal DNA staining was observed in the 1.5-h cocultures with the VCM groups (Fig. S1) but increased following a 3-h incubation in CVVC-R/CD-1 VCM. This was accompanied by PMN clustering with hazy structures similar to the NET-inducing controls. The 1.5 and 3 h coculture with CVVC-S/C3H VCM exhibited some PMN clusters but no hazy appearance around the cells despite low to moderate SYTOX Green staining (Fig. S1). Quantification of fluorescent intensity for all images confirmed a significant reduction in DNA release in the coculture with CVVC-S/C3H VCM compared to those with CVVC-R/CD-1 VCM or the NET-inducing controls (Fig. 1B).

### PMN activation and NET formation are a major driver of antifungal activity in a simulated vaginal environment

**Fig 2:**
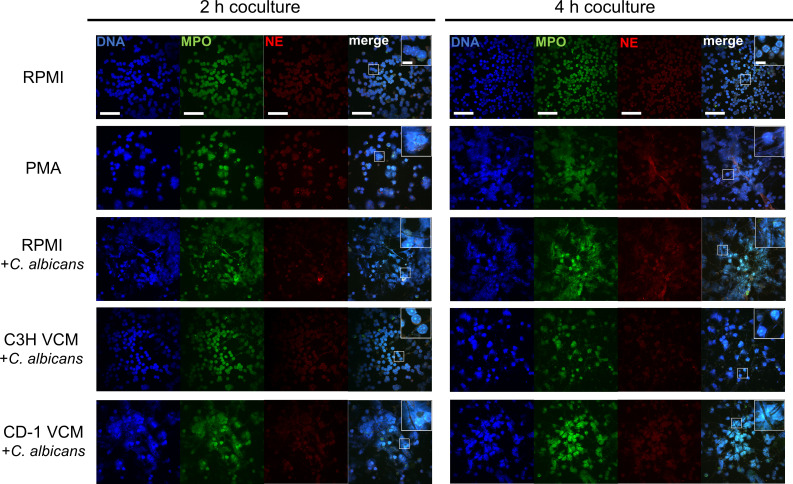
NET formation in the vaginal environment in response to *C. albicans in vitro.* VCM was prepared by pooling vaginal lavage fluid from 5–10 estrogenized C3H (CVVC-susceptible) or CD-1 (CVVC-resistant) mice. (**A**) Monolayers of elicited peritoneal PMNs suspended in VCM or RPMI with PMA cocultured with *C. albicans* 96113 cells for 2 or 4 h and stained with anti-NE (10 µg/mL, red) and anti-MPO (2 µg/mL, green) antibodies followed by Hoechst 33258 (1 µg/mL, blue). Slides were observed by confocal microscopy at a magnification of 600×. Representative areas of color-merged images in white rectangles are magnified and shown in the insets. Scale bars indicate 50 µm (main panels) and 10 µm (insets).

To further evaluate whether PMN antifungal activity is associated with NET-forming capacities in a simulated vaginal environment, PMN*–C. albicans* cocultures under CVVC-susceptible and -resistant conditions were examined for the expression of NE and MPO by immunofluorescent microscopy. Similar to cocultures in NET-inducing conditions in RPMI or with PMA, cocultures in CVVC-R/CD-1 VCM exhibited ruptured cell morphology when in contact with *C. albicans* hyphae after a 2-h incubation. Following 4-h coculture, most of these PMNs further lost cell membrane integrity and showed extruded DNA, with NE and MPO interacting with *C. albicans* hyphae (Fig. 2). In contrast, cocultures in CVVC-S/C3H VCM showed cell morphology of mostly intact PMNs with NE and MPO contained within cells similar to unstimulated PMNs after a 2- or 4-h incubation, with no active interaction with *C. albicans* hyphae (Fig. 2). These results were corroborated by the PMN killing assay confirming a significant reduction in antifungal activity in the cocultures with CVVC-S/C3H VCM, whereas the killing activity in those with CVVC-R/CD-1 VCM was comparable to the NET-inducing controls (Fig. S2).

### NET formation is inhibited by the presence of heparan sulfate in the vaginal environment

**Fig 3:**
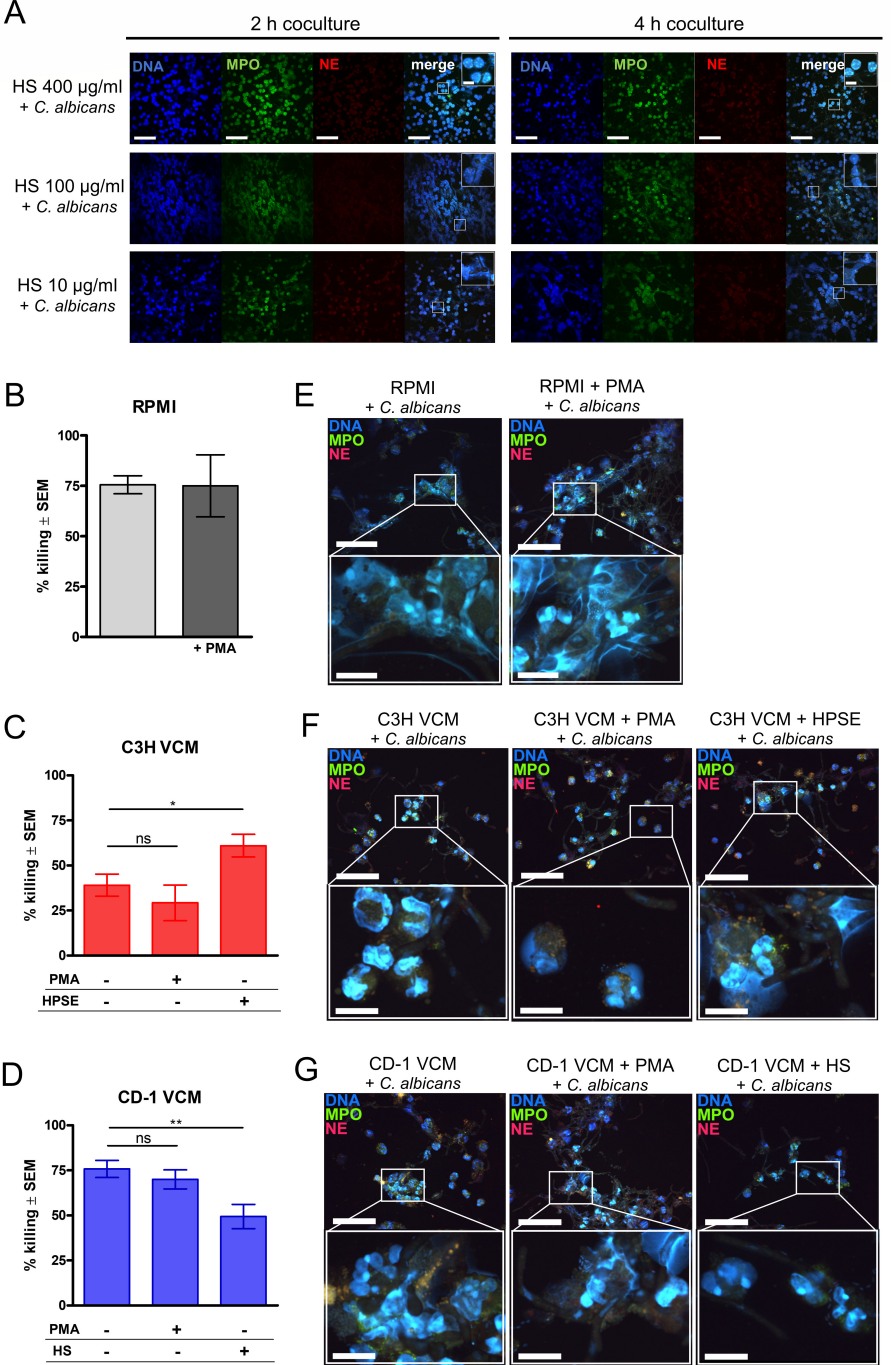
Inhibitory effects of heparan sulfate on NET formation. (**A**) Monolayers of elicited peritoneal PMNs suspended in RPMI medium supplemented with purified heparan sulfate (10, 100, or 400 µg/mL) cocultured with *C. albicans* 96113 cells for 2 or 4 h. Slides were stained with anti-NE (10 µg/mL, red) and anti-MPO (2 µg/mL, green) antibodies overnight at 4°C followed by Hoechst 33258 (1 µg/mL, blue). Slides were observed by confocal microscopy at a magnification of 600×. Representative areas of color-merged images in white rectangles are magnified and shown in the insets. Scale bars indicate 50 µm (main panels) and 10 µm (insets). (**B–D**) Effects of HS and HPSE supplementation on PMN antifungal activity in CVVC-susceptible/C3H and CVVC-resistant/CD-1 VCM. Cocultures of elicited peritoneal PMNs and *C. albicans* 96113 suspended in (**B**) RPMI medium and PMA (100 nM) controls, (**C**) C3H VCM containing PMA or HPSE (5 U), and (**D**) CD-1 VCM containing PMA or purified heparan sulfate (400 µg/mL) were incubated for 3 h and evaluated for *in vitro* killing activity. Viable *C. albicans* cells were enumerated by quantitative plate counts. Data were analyzed using unpaired Student’s *t*-test comparing the medium alone with each supplementation group. Bar heights and error bars reflect the group mean ± SEM of % killing values computed from independent replicates of each of the four unique VCM samples. (**E–G**) Effects of HS and HPSE supplementation on NET formation in CVVC-susceptible/C3H and CVVC-resistant/CD-1 VCM. Monolayers of elicited peritoneal PMNs suspended in VCM or RPMI with PMA cocultured with *C. albicans* 96113 cells for 2 or 4 h and stained with anti-NE (10 µg/mL, red) and anti-MPO (2 µg/mL, green) antibodies followed by Hoechst 33258 (1 µg/mL, blue). Slides were observed by confocal microscopy at a magnification of 600×. Representative areas of the images in white rectangles are magnified and shown in the adjacent panels. Scale bars indicate 50 and 10 µm for the 600× and zoom-in images, respectively. ^*^P < 0.05; ^**^P < 0.01.

Based on our previous report demonstrating vaginal HS as an inhibitory factor of PMN function (16), we sought to investigate whether HS had a similar capacity to reduce NETosis in the CVVC-S condition. For this, we first showed that cocultures of *C. albicans* and PMNs in RPMI medium supplemented with purified HS showed reduced NET formation (Fig. 3A) with confirmatory antifungal activity (Fig. S3) in a dose-dependent manner. Subsequently, a series of mechanistic experiments were conducted to assess whether NET-forming capacities could be modulated by manipulating the vaginal environment under CVVC-S or CVVC-R conditions. PMA supplementation could not reverse the reduced PMN antifungal activity or NET formation in cocultures with CVVC-S/C3H VCM alone (Fig. 3C and F). In contrast, cocultures with CVVC-S/C3H VCM exerted normal fungal killing and NET-forming capacity following pretreatment with heparanase (Fig. 3C and F) at a level similar to the control cocultures using RPMI alone or PMA (Fig. 3B and E). Conversely, the addition of purified HS to cocultures of CVVC-R/CD-1 VCM resulted in a significant inhibition of PMN antifungal activity and NET formation (Fig. 3D and G).

### Vaginal NET-forming capacities in *C. albicans*-inoculated mice susceptible and resistant to vaginitis

**Fig 4:**
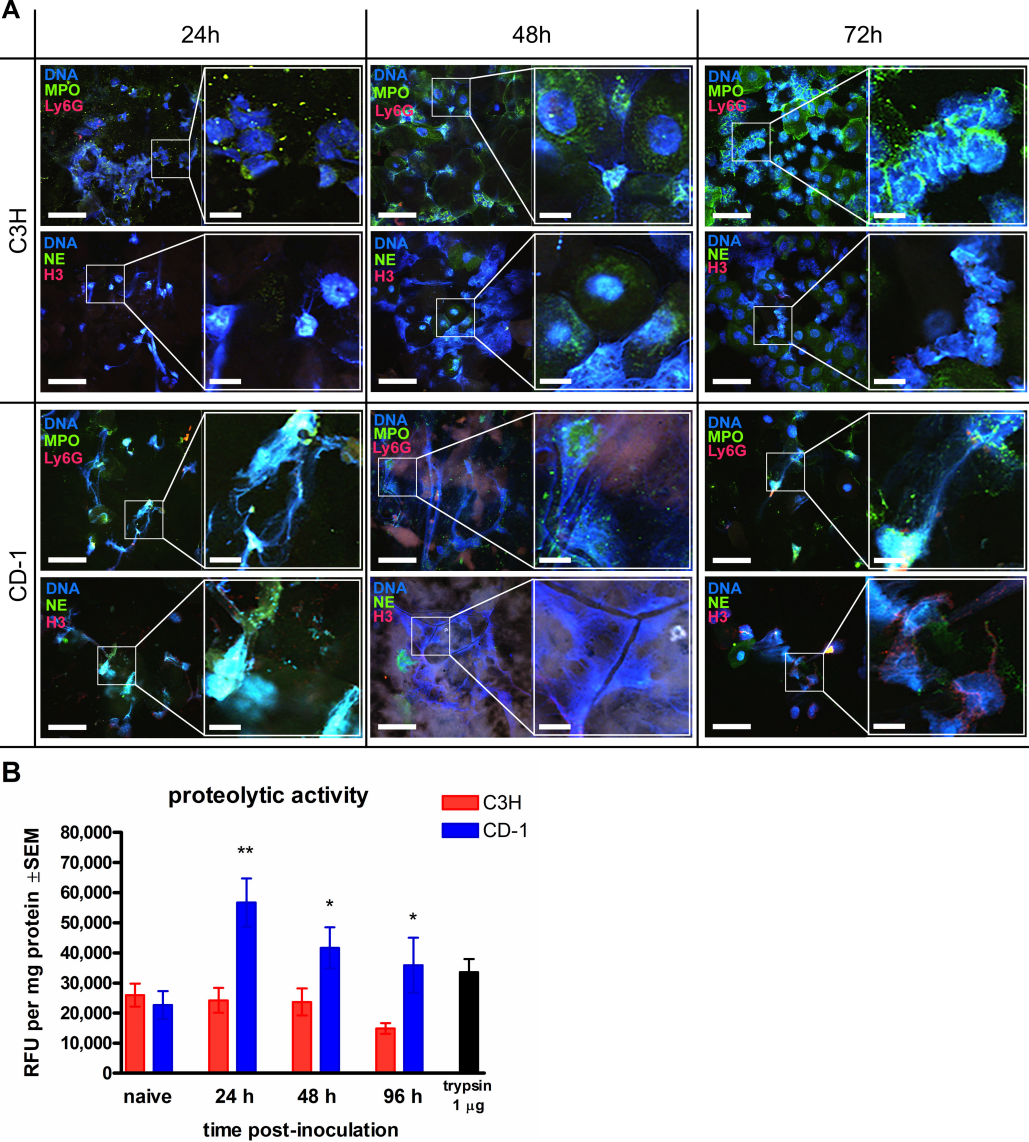
*In vivo* assessment of NET formation in the vaginal cavity following *C. albicans* inoculation of mice susceptible or resistant to CVVC. Estrogenized C3H (CVVC-susceptible) or CD-1 (CVVC-resistant) mice were intravaginally inoculated with *C. albicans* 96113. (**A**) Cytospin preparations of vaginal lavage fluid at 24, 48, and 72 h post-inoculation stained with Hoechst 33342 (1 µg/mL, DNA, blue), anti-MPO (2 µg/mL, green), anti-Ly6G (1 µg/mL, red), anti-NE (10 µg/mL, green), and anti-H3 (5 µg/mL, red) antibodies. Slides were observed by confocal microscopy at a magnification of 600×. Representative areas of the images in white rectangles are magnified and shown in the adjacent panels. Scale bars indicate 50 and 10 µm for the 600× and zoom-in images, respectively. Data represent cumulative results of two independent experiments performed with three to five animals/group. (**B**) Supernatants of vaginal lavage fluid from inoculated mice assessed for relative bioactivity by a proteolytic activity assay. Trypsin (1 µg/mL) was used as a positive assay control. Data were analyzed using unpaired Student’s *t*-test comparing the VCM groups at each time point. Bar heights and error bars reflect the group mean ± SEM of RFU values computed from independent replicates of each of the four unique sets of animals with 5–10 animals/group. ^*^P < 0.05; ^**^P < 0.01; ^***^P < 0.001.

To determine whether resistance and susceptibility to CVVC were associated with observable NET formation and PMN antifungal activity in the vaginal cavity, vaginal lavage fluids from inoculated CVVC-S/C3H and CVVC-R/CD-1 mice were collected longitudinally and analyzed for fungal burden, NET formation, and proteolytic activity of vaginal secretions. Consistent with previous reports, quantification of vaginal fungal burden by CFU counts confirmed a steady decline followed by clearance within 10–14 days post-inoculation in all CVVC-R/CD-1 mice, while CVVC-S/C3H mice sustained vaginal colonization (Fig. S4). Vaginal lavage samples examined for the presence of NETs showed that in both samples from CVVC-S/C3H mice and CVVC-R/CD-1 mice, an initial PMN response to *C. albicans* was observed at 24 h post-inoculation that continued at 48 and 72 h post-inoculation. However, the PMNs from CVVR-S/C3H mice had largely intact cell morphology with DNA and cytosolic proteins within the cells, whereas PMNs from CVVC-R/CD-1 mice showed a heterogenous infiltrate of intact and NETotic cells as identified by extruded DNA colocalized with MPO, NE, Ly6G, and histone (H3) (Fig. 4A). To assess relative bioactivity of vaginal secretory factors associated with NETs, supernatants of vaginal lavage fluids were evaluated by a proteolytic activity assay. Results showed that vaginal secretions from CVVC-R/CD-1 mice exerted substantial proteolytic activity at 24 h post-inoculation followed by a gradual decrease after 48 and 96 h post-inoculation (Fig. 4B). In contrast, significantly reduced activity was evident in vaginal secretions from CVVC-S/C3H mice at all time points.

### Induction of NETosis via Ki67 is associated with resistance to CVVC

**Fig 5:**
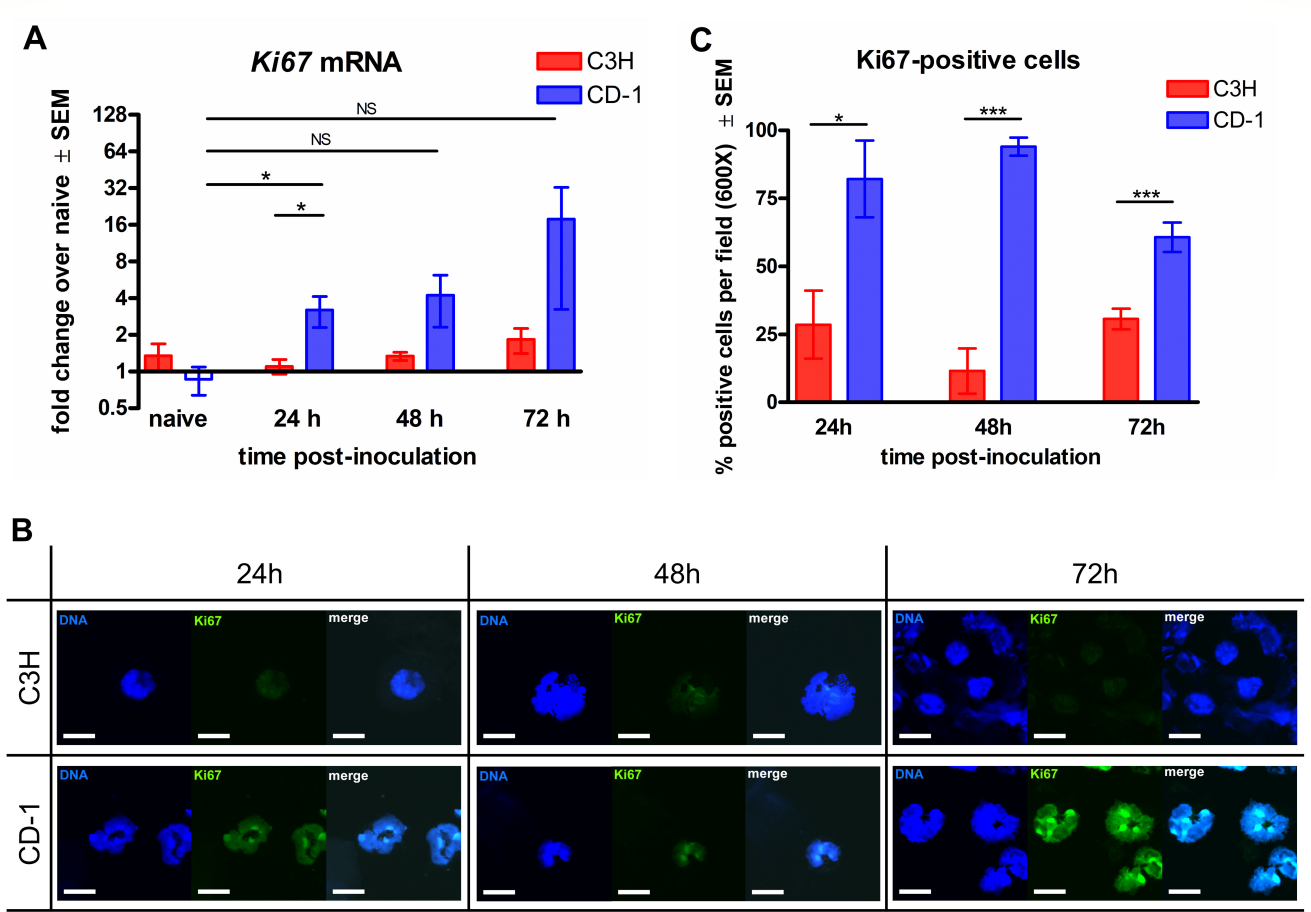
NET-inducing capacity of PMNs by Ki67 expression in response to *C. albicans* vaginal colonization. Estrogenized C3H (VVC-susceptible) or CD-1 (VVC-resistant) mice were intravaginally inoculated with *C. albicans* 96113, and vaginal lavage fluid was collected daily from 0, 24, 48, and 72 h post-inoculation. (**A**) RNAs isolated from the cell fraction of vaginal lavage fluids analyzed for *Ki67* expression by qPCR. (**B**) Cytospin preparations of vaginal lavage fluid stained with Hoechst 33342 (1 µg/mL, DNA, blue) and anti-Ki67 (5 µg/mL, green) antibodies and observed by confocal microscopy at a magnification of 600× and further magnified to a 3,000× final magnification. Scale bars indicate 10 µm. Data represent cumulative results of two independent experiments performed with three to five animals/group. (**C**) The percentage of Ki67-positive cells was quantified among the total PMN population within the microscopic field observed at 600× magnification. PCR (**A**) and cell quantification data (**C**) were analyzed using unpaired Student’s *t*-test comparing the two mouse strains at each time point. Bar heights and error bars reflect the group mean ± SEM of the values computed from independent replicates of each of three to four unique sets of animals with 5–10 animals/group. ^*^P < 0.05; ^**^P < 0.01; ^***^P < 0.001.

Previous studies have demonstrated the striking similarities between the processes of mitosis in dividing cells and NETosis in terminally differentiated PMNs (27). Notably, a cell cycle regulator Ki67 was found to be upregulated in PMNs undergoing NETosis and has been suggested to serve as a maker of NETotic PMNs. To further verify that NET formation contributes to fungal clearance during vaginitis, cell fractions of vaginal lavage fluids from CVVC-R/CD-1 mice and CVVC-S/C3H mice were examined for *Ki67* expression. Gene expression analysis by quantitative polymerase chain reaction (qPCR) showed that levels of *Ki67* mRNA in vaginal cells from CVVC-R/CD-1 mice were significantly increased at 24 h post-inoculation, whereas no upregulation of *Ki67* was observed in cells from CVVC-S/C3H mice (Fig. 5A). Although there was a consistent trend of increased Ki67 expression at subsequent time points, no statistical difference was achieved due to intragroup variabilities (CD-1: *P* = 0.086 and *P* = 0.30, C3H: *P* = 0.98 and *P* = 0.39, at 48 and 72 h post-inoculation, respectively). As additional confirmation of these findings, PMNs from vaginal lavage fluids were stained with anti-Ki67 antibodies and evaluated for protein expression within the nucleus. Visualization by immunofluorescence displayed colocalization of Ki67 and nuclear staining in the majority of PMNs with intact nuclei from CVVC-R/CD-1 mice at 24–72 h post-inoculation (Fig. 5B). In contrast, Ki67 staining was largely absent or scarce in PMNs from CVVC-S/C3H mice at all time points (Fig. 5B). Quantification of the staining data confirmed that the percentage of nuclear-intact/Ki67-positive PMNs (i.e., NET precursors) was significantly higher in the CVVC-R/CD-1 group compared to the CVVC-S/C3H group (Fig. 5C).

### NET formation is visually impaired in CVVC-susceptible conditions *in vitro* and *in vivo*

**Fig 6:**
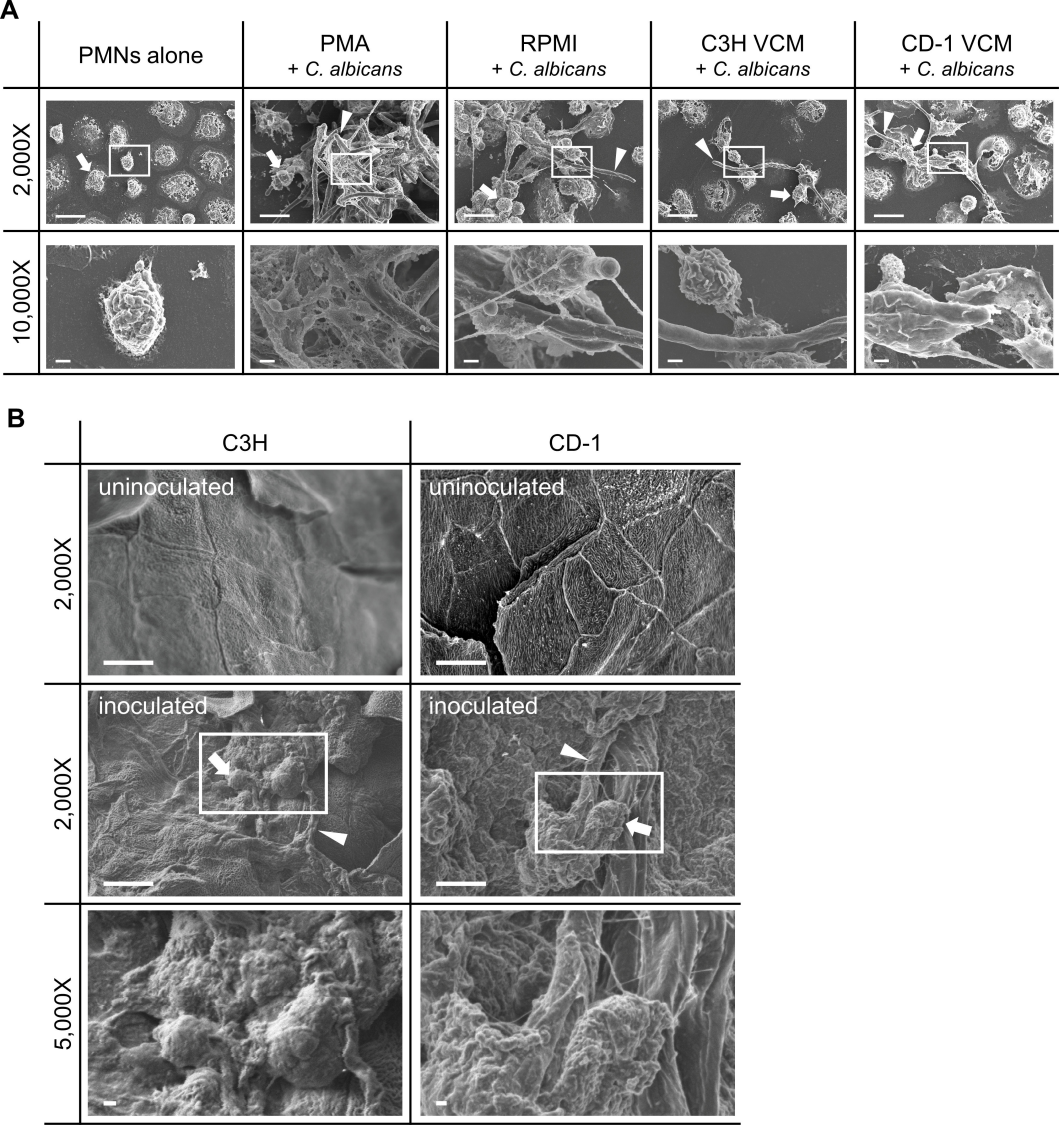
NET-inducing capacity of PMNs in response to *C. albicans in vitro* and *in vivo* by SEM. VCM was prepared by pooling vaginal lavage fluid from 5–10 estrogenized C3H (CVVC-susceptible) or CD-1 (CVVC-resistant) mice. (**A**) SEM images of elicited peritoneal PMNs suspended in VCM or RPMI with PMA. PMNs were seeded onto coverslips in 24-well plates and either cultured alone or cocultured with *C. albicans* 96113 cells for 3 h. (**B**) SEM images of vaginal tissues from estrogenized C3H (CVVC-susceptible) or CD-1 (CVVC-resistant) mice inoculated with *C. albicans* 96113 for 48 h. The coverslips collected from the cocultures (**A**) and resected tissues in 4% paraformaldehyde were processed for SEM. Representative areas of the images in white rectangles are magnified and shown in the lower panels. Arrows and arrowheads indicate PMNs and *C. albicans*, respectively. Scale bars indicate 10 µm at 2,000× and 1 µm at 10,000× (**A**) and 5,000× (**B**) magnifications. Coculture data represent two independent experiments performed with two unique sets of VCM samples (**A**). Data involving vaginal tissues represent two independent experiments performed using five mice/group (**B**).

To further validate the distinct NET-forming capacities under CVVC-susceptible and -resistant conditions, SEM was employed to visualize NETs both *in vitro* and *in vivo*. In *in vitro* cocultures with CVVC-R/CD-1 VCM, PMNs exhibited web-like structures consisting of extruded DNA fibers in direct contact with *C. albicans* hyphae (Fig. 6A), similar to those observed in cocultured in RPMI or PMA-stimulated controls. In contrast, PMNs in CVVC-S/C3H VCM did not show the characteristic NETotic phenotype despite the presence of *C. albicans* hyphae (Fig. 6A). Similar results were observed in cocultures with both VCM groups using *C. albicans* SC5314 (data not shown). *In vivo* analyses showed that vaginal tissues from CVVC-R/CD-1 mice 48 h post-inoculation had considerable NET formation on the epithelial surfaces, composed of extension of extruded DNA fibers interacting with *C. albicans* hyphae (Fig. 6B), whereas vaginal tissues from CVVC-S/C3H mice exhibited fungal biofilm growth on the epithelial surfaces, comprised of *C. albicans* hyphae embedded in extracellular matrices with no evidence of NETs (Fig. 6B).

## DISCUSSION

Despite the wide availability of antifungal treatments, the incidence of VVC/RVVC has remained high and unchanged for several decades (5). The inability of current antifungal therapies to provide complete cure or long-term symptom relief poses considerable challenges in affected women, leading to prolonged healthcare burden and diminished quality of life. Given that competent PMNs are robustly recruited into the vagina without exerting appreciable antifungal activity, we hypothesized that impaired formation of NETs, a crucial PMN defense mechanism implicated in various candidiasis models (22, 33–35), is a major piece of the dysfunction that contributes to the lack of fungal clearance during vaginitis.

The protective role of NETs against fungal pathogens is well established in various mucosal and invasive infections. Studies have demonstrated the ability of NETs to capture and kill *C. albicans* hyphae in *in vitro* coculture settings as well as different anatomical sites *in vivo*, including kidneys, peritoneum, and lung (22, 35, 36). However, the specific involvement of NETs in the vaginal compartment has not been intensely investigated. The only reports to date include one study showing significant antimicrobial capacity by NETs against *Trichomonas vaginalis* in human neutrophils (37) and a clinical study reporting increased NETs in vaginal discharges of women with *T. vaginalis* vaginitis (31). Interestingly, this same study indicated that NETs were found but only marginally increased in samples from symptomatic VVC women. The small sample size, however, precluded definitive conclusions for any direct contribution of NETs, or lack thereof, for either infection. Here, with the use of an established mouse model incorporating chronic VVC-susceptible (CVVC-S/C3H) and VVC-resistant (CVVC-R/CD-1) mice, our findings suggest a strong association between NETs and resistance, but not susceptibility, to *C. albicans* vaginitis.

Our *in vitro* experiments using VCM simulating CVVC-S and CVVC-R conditions revealed little to no NET formation under the CVVC-S condition in response to *C. albicans*, whereas PMNs underwent substantial NETosis in the CVVC-R condition. Importantly, the divergent outcomes in NET formation correlated with those observed for PMN antifungal activity. Consistent with previous reports, this inhibitory effect on NET formation and PMN activity was reproducible by the addition of HS, the competitive inhibitor of PMN*–Candida* interactions in the vagina that promotes the susceptible condition in the mouse model (16, 38). Importantly, pretreatment of the CVVC-S/C3H VCM with heparanase resulted in the restoration of the ability for both NET formation and fungal killing, further supporting the inhibitory effect of HS on PMN antifungal activity through NETs. Interestingly, the lack of antifungal activity was sustained in PMNs pretreated with PMA in the CVVC-S VCM coculture. Hence, the diminished antifungal activity in the vaginal environment cannot be rescued simply by stimulating PMNs through the use of NET-inducing PMA. Also unexpected was a less pronounced inhibitory effect of HS in the reverse setting where cocultures with CVVC-R/CD-1 VCM were supplemented with HS, compared to the strong inhibition observed when cocultures with RPMI alone were supplemented with HS (Fig. S3). Given the complex vaginal environment where a multitude of secretory factors from both the host and microbes are present, we hypothesize that the abundance of such local factors could potentially interfere with the efficacy of certain exogenous agents. While heparan sulfate is found in various tissues including the vaginal mucosa (39, 40), the specific concentrations of heparan sulfate in vaginal secretions of mice and women remain unclear largely due to the lack of reliable immunoassays. Further optimization is needed for *in vivo* exploration.

In the subsequent series of *in vivo* experiments, the association between susceptibility/resistance to VVC and the expression of Ki67, a protein involved in cell cycle signaling and implicated in NETotic PMNs, was explored. Work by Amulic et al. reported a remarkable discovery that PMNs, which are terminally differentiated, utilize proteins of the cell division system to initiate processes of NETosis (27). These events shared between mitosis and NETosis include nuclear envelope disintegration that is active when NETosis is triggered in PMNs. The significant upregulation of Ki67, confirmed by mRNA expression and immunofluorescence in PMNs from inoculated CVVC-R mice, indicates active NET induction. In contrast, PMNs from CVVC-S mice exhibited minimal Ki67 expression, further supporting impaired NET formation under the CVVC-S condition and its potential contribution to diminished antifungal activity and fungal persistence in the vaginal environment of susceptible hosts.

The importance of NETs in effective clearance of *C. albicans* during vaginitis is further supported by the observation that vaginal secretions from CVVC-R mice exhibited substantial proteolytic activity. Studies examining the antimicrobial activity of NETs have demonstrated that proteolytic enzymes released by NETs, such as neutrophil elastase and lactoferrin, exhibited bactericidal activity and can degrade *Staphylococcus aureus* biofilms (41, 42). However, we recognize that not all NET-derived antimicrobial enzymes have direct fungicidal effects, and some mainly act as chelators of metal ions required for microbial survival (22, 43). Accordingly, we postulate that the observed proteolytic activity contributes to the inhibition of *C. albicans* colonization/biofilm formation on the vaginal epithelium by disrupting the development of the extracellular matrix (ECM) necessary for mature biofilm growth. This is supported by the visual examination of vaginal tissues from inoculated CVVC-S and CVVC-R mice via SEM, the former exhibiting fungal biofilm growth on the vaginal epithelium in line with previous reports (44), while the latter showed NETs in direct contact with *C. albicans* hyphae and minimal evidence of biofilms. Interestingly, studies by Johnson et al. have indicated that *C. albicans* biofilms can modulate NET release through ECM-induced inhibitory pathways (33), suggesting a potential negative feedback regulation dependent on the extent of biofilm growth. Collectively, our data strongly suggest that CVVC-S mice present with a biofilm-associated infection phenotype devoid of any appreciable biofilm reduction/inhibition by NETs in addition to reduced antifungal activity.

Despite the use of a well-established VVC mouse model, we can appreciate potential limitations in investigating NET formation in these particular strains of mice. Notably, CVVC-R/CD1 mice have inherent hypo-responsiveness to exogenous estrogen and, thus, do not respond to the exogenous estrogen administration at the dosing required for sustained vaginal fungal colonization (45, 46). Several studies evaluating the possible effects of 17-β-estradiol, or its target receptors, have reported both reducing or enhancing potentials for NET-forming capacities (47, 48). In either case, however, our model, which requires a continuous pseudoestrus state, would preclude a reliable validation of the estrogen-dependent effects on NETosis. Building on this notion, we also recognize a limitation in this study focusing on the two strains, inbred C3H and outbred CD-1 mice exclusively. While previous studies have shown comparable CVVC-S outcomes in various other inbred strains (14), it is important to acknowledge that drawing conclusions based solely on comparisons between the two mouse strains may not be directly translatable to clinical scenarios. Instead, the model was utilized to explore the mechanisms of immune resistance/clearance and susceptibility/pathologic responses, which are observed similarly in both mice and humans (13). Accordingly, *C. albicans* 96113, a vaginal isolate, was chosen for use in this study to enhance clinical relevance. However, we recognize that results may differ with other strains. Another noteworthy limitation of this model pertains to the notion that, contrary to the widely accepted concept of hyphae to induce NET formation, the capacity of yeast to induce NET formation remains controversial. Specifically, there is some evidence supporting the involvement of NET release in response to non-*Candida albicans* spp. including *Candida glabrata* (49). However, since the murine model of *C. glabrata* vaginitis shows no immunopathologic response via PMN infiltration (50, 51), the model is not suitable to address this question.

Finally, it is noteworthy that, while NETs appear to play a protective or resistant role in controlling *C. albicans* infections, excessive or dysregulated NET formation can lead to tissue damage and deleterious inflammation. The literature provides accumulating evidence of NET-induced pathologies across various infectious diseases, autoimmune diseases, preterm birth, sepsis, and tumor metastasis (52–56). Hence, caution should be exercised when manipulating/inducing NET responses as excessive NETs could inadvertently exacerbate existing damage that occurs in the absence of NETs (i.e., CVVC-S mice).

Our results may provide novel insights into strategies for restoring vaginal homeostasis. One strategy is to inhibit PMNs from infiltrating the vaginal cavity altogether. While this approach would reduce the deleterious inflammation, it would concurrently allow *C. albicans* hyphal growth to persist. Moreover, a lack of PMNs would hinder their defense against other potential pathogens present in the vaginal cavity. Ensuing overgrowth of microorganisms could potentially trigger secondary PMN migration to a greater extent once the native cellular migration capacity is restored. A second strategy would be to neutralize local HS thereby enabling migrating PMNs to exert antifungal activity in the vaginal cavity. This would be the more ideal strategy that could promote fungal clearance. Unfortunately, attempts in the animal model to either inhibit PMN migration or block/eliminate HS have been unsuccessful to date. Recently, there are some data showing that zinc can effectively inhibit PMN migration *in vitro* and *in vivo*, by binding to and potentially modulating *C. albicans* pH-regulated antigen 1 (Pra1) protein (57). Interestingly, this same study reported that intravaginal zinc treatment reduced the number of recurrences in women with RVVC, presumably due to dampened Pra1-mediated PMN migration (57). More clinical studies will be necessary to confirm a definitive anti-inflammatory role for zinc. As for the alternate strategy of neutralizing HS, strong clinical evidence of vaginal HS in cases of VVC/RVVC is still needed. Current studies in our laboratory are underway to address this important question.

In conclusion, our findings reveal disparate NET-forming capacities between two mouse strains that represent resistance and susceptible conditions of chronic VVC. Impaired NET formation is associated with fungal persistence in CVVC-S/C3H mice, while effective NET formation in CVVC-R/CD-1 mice leads to fungal clearance. Consequently, NETosis appears to play a crucial role in protection against *C. albicans* at the vaginal mucosa. The intricate balance between NET-mediated protection and PMN-driven immunopathology in VVC/RVVC pathogenesis remains an intriguing area of active research. Further research is needed to elucidate the specific molecular mechanisms underlying NET induction, regulation, and impact on resistance to infection.

## SUPPLEMENTAL MATERIAL

Figure S1 iai.00350-23-s0001.tif Visualization of DNA release by PMN-*C. albicans* cocultures under in CVVC-susceptible and CVVC-resistant conditions in vitro.

Figure S2 iai.00350-23-s0002.tif Antifungal activity of PMNs in CVVC-susceptible and CVVC-resistant VCM.

Figure S3 iai.00350-23-s0003.tif Inhibitory effects of heparan sulfate on antifungal activity in vitro.

Figure S4 iai.00350-23-s0004.tif Vaginal fungal burden following *C. albicans* inoculation of mice susceptible or resistant to CVVC.

Supplemental text iai.00350-23-s0005.docx Supplemental methods, and supplemental legends for Fig. S1 to S4.

ASM does not own the copyrights to Supplemental Material that may be linked to, or accessed through, an article. The authors have granted ASM a non-exclusive, world-wide license to publish the Supplemental Material files. Please contact the corresponding author directly for reuse.
