# Supplementary figures and images for: Impaired neutrophil extracellular trap-forming capacity contributes to susceptibility to chronic vaginitis in a mouse model of vulvovaginal candidiasis
Source: Infect Immun. 2024 Jan 30;92(3):e00350-23. doi: 10.1128/iai.00350-23 (PMC10929430; doi:10.1128/iai.00350-23)

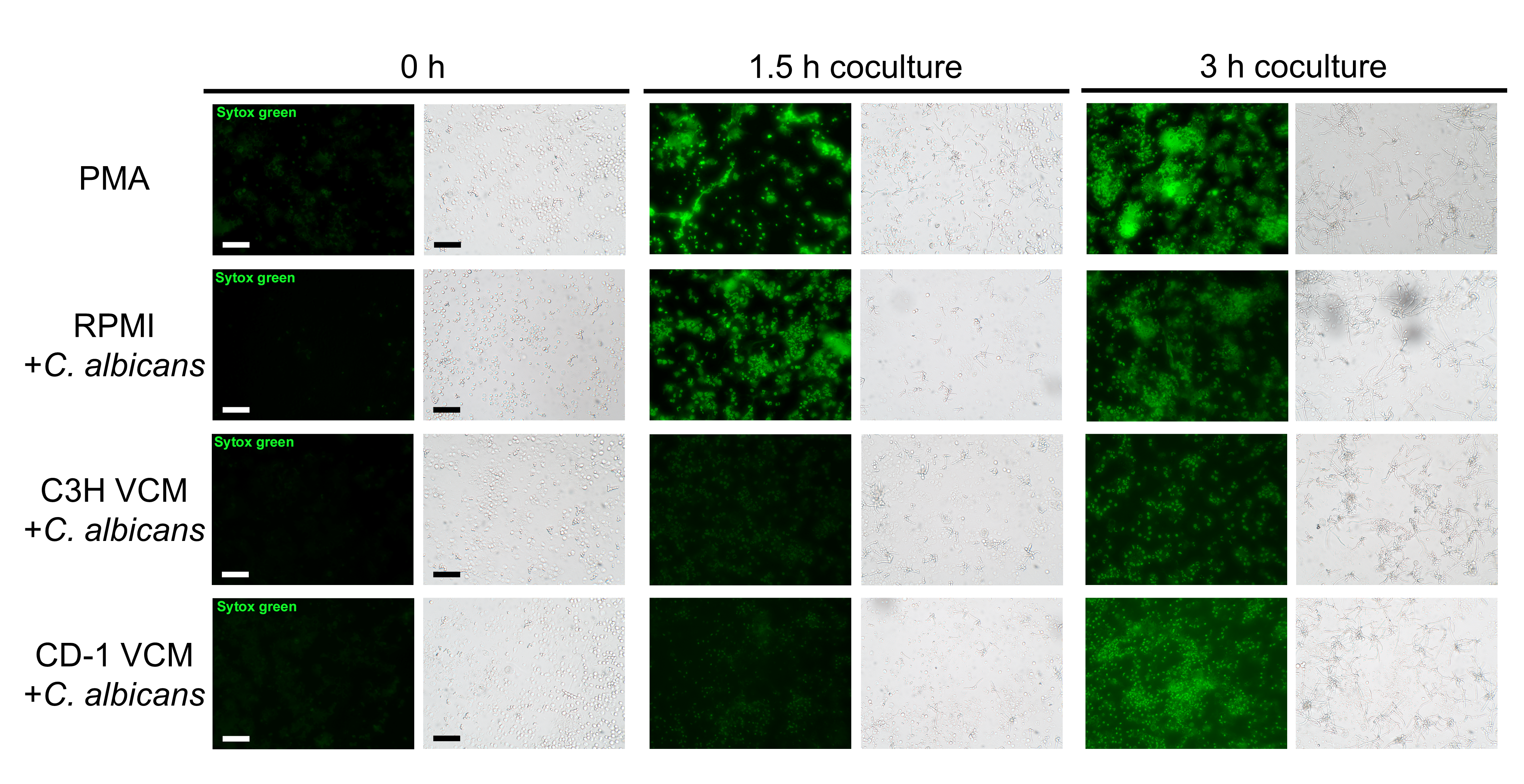

Supplement: Figure S1 — Visualization of DNA release by PMN-C. albicans cocultures under in CVVC-susceptible and CVVC-resistant conditions in vitro. [file iai.00350-23-s0001.tif]

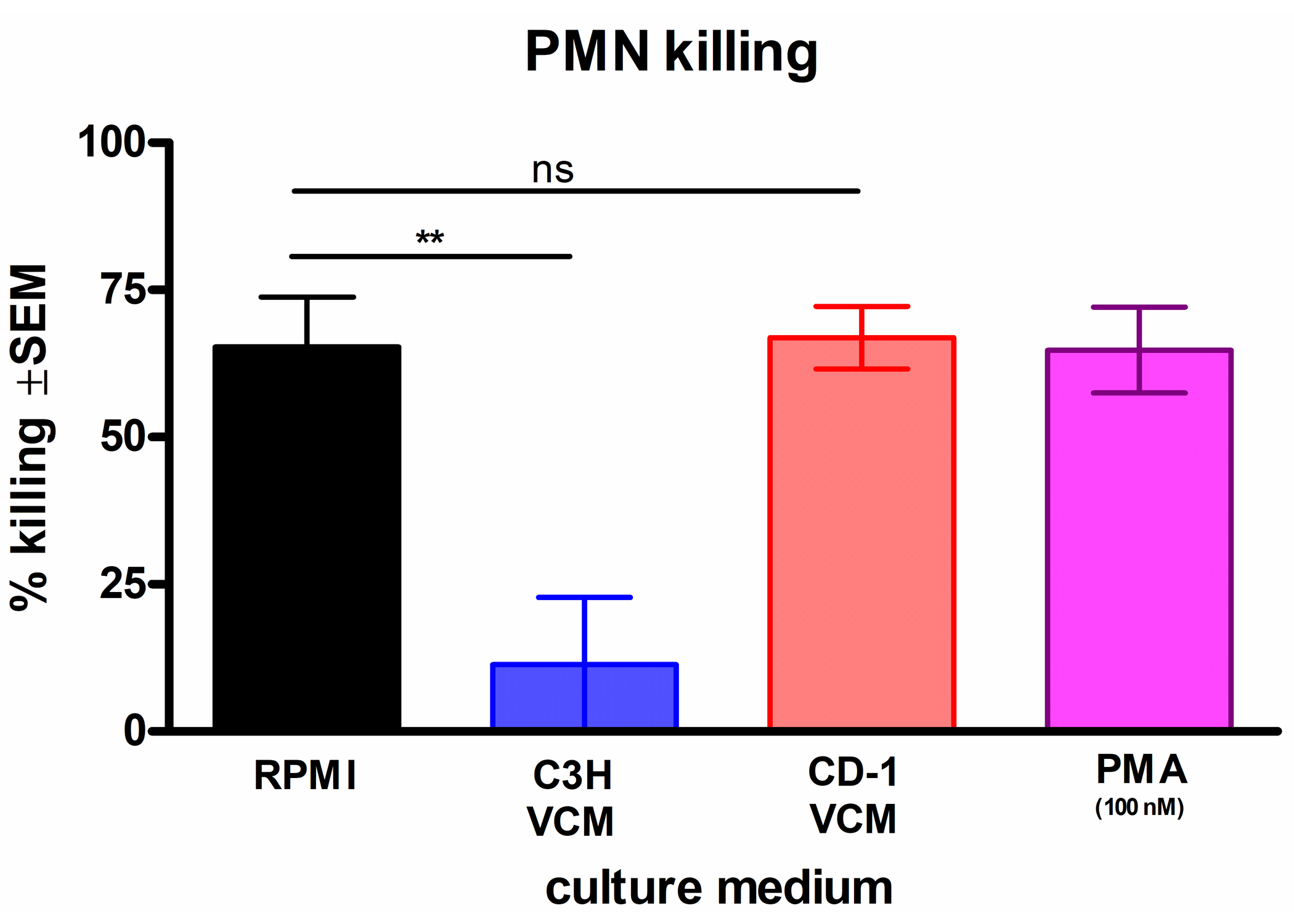

Supplement: Figure S2 — Antifungal activity of PMNs in CVVC-susceptible and CVVC-resistant VCM. [file iai.00350-23-s0002.tif]

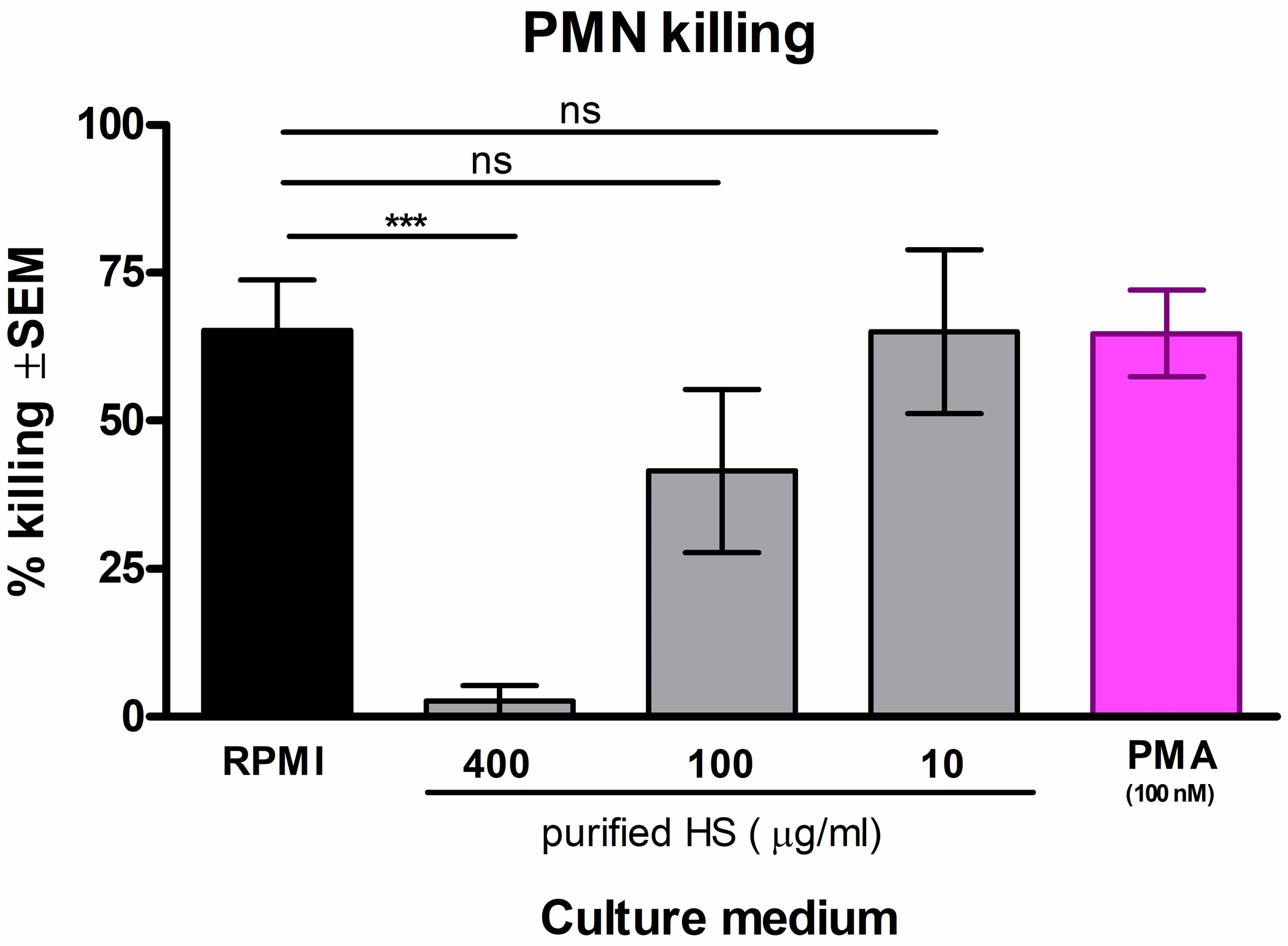

Supplement: Figure S3 — Inhibitory effects of heparan sulfate on antifungal activity in vitro. [file iai.00350-23-s0003.tif]

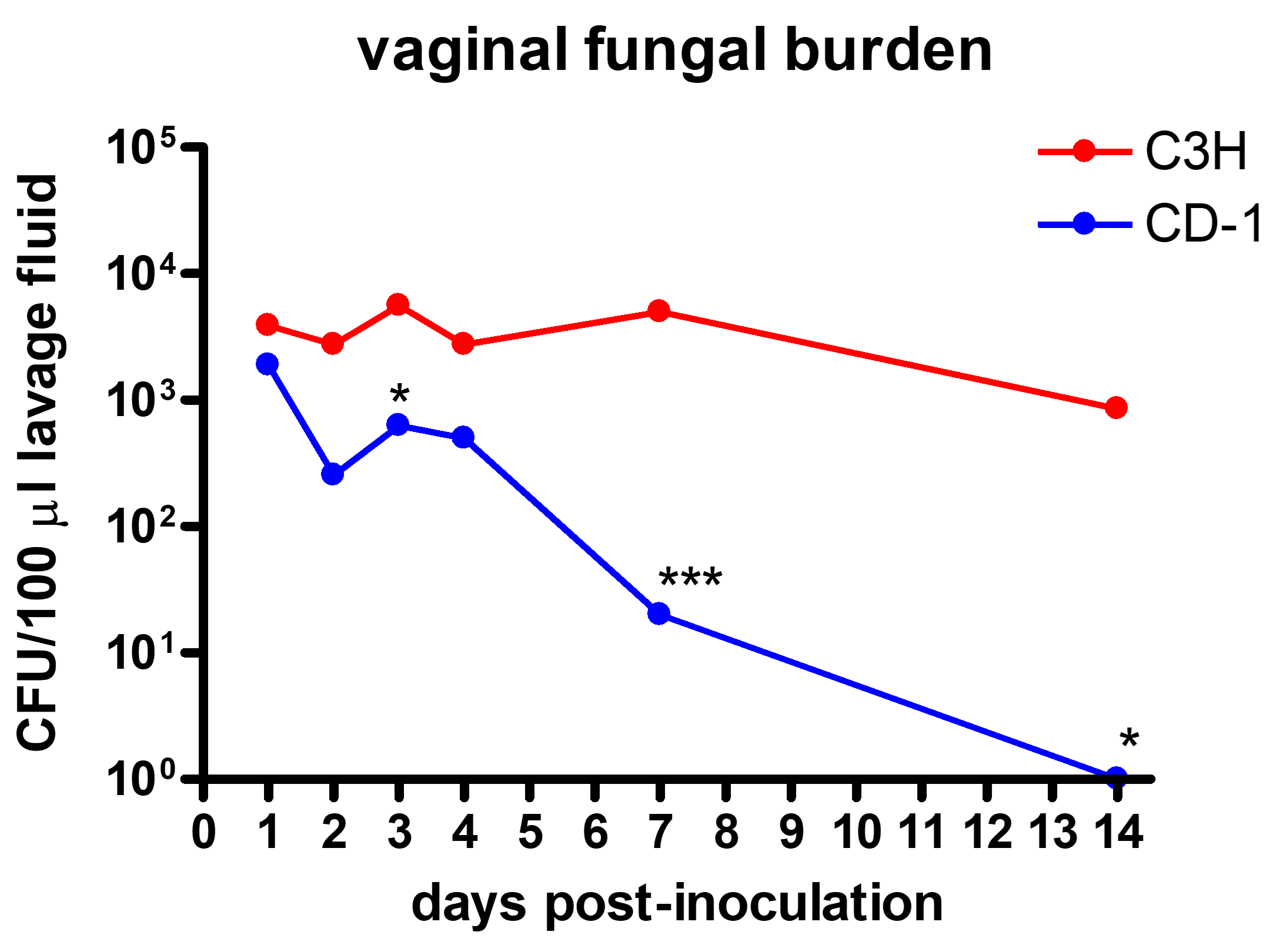

Supplement: Figure S4 — Vaginal fungal burden following C. albicans inoculation of mice susceptible or resistant to CVVC. [file iai.00350-23-s0004.tif]
