# Supplementary material for: Impaired neutrophil extracellular trap-forming capacity contributes to susceptibility to chronic vaginitis in a mouse model of vulvovaginal candidiasis
Source: Infect Immun. 2024 Jan 30;92(3):e00350-23. doi: 10.1128/iai.00350-23 (PMC10929430; doi:10.1128/iai.00350-23)
Supplement: Supplemental text — Supplemental methods, and supplemental legends for Fig. S1 to S4. [file iai.00350-23-s0005.docx]

**Supplemental Materials and Methods**

**Visualization of extracellular DNA release.** Cocultures of PMN (5 x 10^5^/well) and *C. albicans* (5 x 10^4^/well) in a volume of 100 µl were prepared in a 96-well plate using RPMI 1640 alone or experimental VCM in the presence of cell-impermeable Sytox Green DNA-binding dye (5 µM) and incubated for 3 h at 37°C with 5% CO_2_. During the incubation, the plate was transferred onto a Nikon Eclipse E200 fluorescent microscope at 0, 1.5 and 3 h time points without permeabilization and observed at a 20X magnification.

**Assessment of vaginal fungal burden.** Vaginal lavages were conducted in inoculated mice at specific time points 1-14 days post-inoculation. Aliquots of lavage fluids were serially diluted and cultured on Sabouraud-dextrose agar plates supplemented with gentamicin to assess vaginal fungal burden. Colony-forming unites (CFUs) were enumerated after incubation for 24 h at 35°C and expressed as CFU/100 µl of lavage fluid.

**Supplemental figure legends**

**Supplemental figure 1. Visualization of DNA release by PMN-*C. albicans* cocultures under in CVVC-susceptible and CVVC-resistant conditions *in vitro*.** Vaginal conditioned medium (VCM) was prepared by pooling vaginal lavage fluid from 5 to 10 estrogenized C3H (CVVC-susceptible) or CD-1 (CVVC-resistant) mice. Elicited peritoneal PMNs were suspended in VCM or RPMI with PMA, pre-incubated to form monolayers, and cocultured with *C. albicans* 96113 cells for 3 h in the presence of cell-impermeable Sytox Green DNA-binding dye (5 µM). At indicated time points, the plate was observed under FITC or brightfield filters at a 200X magnification. Scale bars indicate 100 µm. Data represent cumulative results of 4 independent experiments performed with 4 unique sets of VCM samples. PMA*,* Phorbol 12*-*myristate 13*-*acetate; PMN, polymorphonuclear leukocyte; VCM, vaginal conditioned medium.

**Supplemental figure 2. Antifungal activity of PMNs in CVVC-susceptible and CVVC-resistant VCM.** Cocultures of elicited peritoneal PMNs and *C. albicans* 96113 were prepared in a 96 well plate, incubated for 3 h and examined for *in vitro* killing activity. Viable *C. albicans* cells were enumerated by quantitative plate counts. Cocultures in RPMI alone or with PMA (100 nM) were tested in parallel as positive controls. Data were analyzed using the One-Way ANOVA among the RPMI control and the VCM groups followed by the unpaired Student’s *t* test to compare each VCM group with the control. Bar heights and error bars reflect group mean ± standard error of the mean (SEM) of % killing values computed from independent replicates of each of the three unique VCM samples. **, *P* < 0.01; n.s, not significant; PMA*,* Phorbol 12*-*myristate 13*-*acetate; PMN, polymorphonuclear leukocyte; VCM, vaginal conditioned medium.

**Supplemental figure 3. Inhibitory effects of heparan sulfate on antifungal activity *in vitro.***  Cocultures of elicited peritoneal PMNs and *C. albicans* 96113 suspended in RPMI medium containing purified heparan sulfate (10, 100, 400 µg/ml) were incubated for 3 h and evaluated for *in vitro* killing activity. Cocultures in RPMI alone or with PMA (100 nM) were tested in parallel as positive controls. Viable *C. albicans* cells were enumerated by quantitative plate counts. Data were analyzed using the unpaired Student’s *t* test comparing the RPMI control and each HS group. Bar heights and error bars reflect group mean ± standard error of the mean (SEM) of % killing values computed from independent replicates of each of the three unique HS preparations. ***, *P* < 0.001; n.s, not significant; PMA*,* Phorbol 12*-*myristate 13*-*acetate; PMN, polymorphonuclear leukocyte; HS, heparan sulfate.

**Supplemental figure 4. Vaginal fungal burden following *C. albicans* inoculation of mice susceptible or resistant to CVVC.** Estrogenized C3H (VVC-susceptible) or CD-1 (VVC-resistant) mice were intravaginally inoculated with *C. albicans* 96113 and vaginal lavage fluid was collected from 1-14 days post-inoculation. Vaginal fungal burden was assessed by quantitative plate counts. Data were analyzed using the Mann-Whitney *U* test comparing the two mouse strains at each time point. Data represent cumulative results of 3 independent experiments performed with 5-10 animals/group. *, *P* < 0.05; ***, *P* < 0.001; CFU*,* colony forming unit.
